# Anti cancer activity of colon specific osmotic drug delivery of acetone extract of Quercus Infectoria Olivier, Fagaceae in 1,2-dimethylhydrazine-induced colon cancer in rats

**DOI:** 10.1186/1755-8166-7-S1-P14

**Published:** 2014-01-21

**Authors:** Roshni Solanki, Dhaval Madat, Ramesh K Goyal

**Affiliations:** 1 Faculty of Pharmacy, Dharmsinh Desai University, Nadiad, India; 2 TBC; 3 Institute of Life Science, Ahmedabad University, Ahmedabad, India

## Background

Adenoma of colon and rectum is known as colorectal cancer. It is the fourth most common form of cancer in the United States. Various colon drug targeting systems are currently under development to minimize drug degradation, prevention of side-effects and increase drug bioavailability in the required zone. Galls of Q.infectoria contains about 50%-70% tannin mainly gallotannic acid which is reported to be antimutagenic. In present investigation colon targeted AEQI formulation was prepared and evaluated by in vitro drug release study and in vivo study using 1,2-dimethylhydrazine(DMH) induced colon cancer in rats with or without diabetes.

## Materials and methods

Pellets of AEQI were prepared by extrusion-spheronization method using Chitosan as osmotic agent. Optimized formulation was tested further for in vitro study, stability study and release kinetic model fitting study. In vivo study was carried out using 1,2-dimethylhydrazine (DMH) 20 mg/kg s.c. in rats. Diabetes was induced by streptozotocin 40 mg/kg i.v. Colon targeted formulation of AEQI of 100mg/kg and 200 mg/kg was administered for 16 weeks. At the end of study blood samples were collected for estimation of antioxidant and oxidant parameters and measurement of TNF α and TGF β levels. Colon was isolated at the end of study and development of aberrant crypt foci (ACF) was observed in histopathology and tissue VEGF levels were measured. Statistical analysis was carried out using analysis of variance followed by multiple comparison Tukey (p<0.05).

## Results

In vitro study showed that optimization gave sustained release of drug for 24 hr 98.37% release and stability with respect to release pattern. The release of drug follows Weibull model with minimum F value (22.40). In vivo treatment showed improvement in oxidant and antioxidant parameters significantly with improved histopathological characters in colon as compared to non treated model control group. Moreover significant reduction in TNF α, TGF β and VEGF levels was observed with treatment. Diabetes associated with colon cancer increases severity of colon cancer and it was prevented by treatment.

## Conclusions

Our data suggest that microbially triggered colon specific osmotic pump delivery have a potential to be used as targeted therapy for cancer.

